# Genome-Wide Analysis of PL7 Alginate Lyases in the Genus *Zobellia*

**DOI:** 10.3390/molecules26082387

**Published:** 2021-04-20

**Authors:** Nadezhda Chernysheva, Evgeniya Bystritskaya, Galina Likhatskaya, Olga Nedashkovskaya, Marina Isaeva

**Affiliations:** G.B. Elyakov Pacific Institute of Bioorganic Chemistry, Far Eastern Branch, Russian Academy of Sciences, 159, Pr. 100 let Vladivostoku, 690022 Vladivostok, Russia; chernysheva.nadezhda@gmail.com (N.C.); belyjane@gmail.com (E.B.); galin56@mail.ru (G.L.); oned2004@mail.ru (O.N.)

**Keywords:** *Zobellia*, genomes, polysaccharide lyase family 7, alginate utilization system, paralogs, orthologs

## Abstract

We carried out a detailed investigation of PL7 alginate lyases across the *Zobellia* genus. The main findings were obtained using the methods of comparative genomics and spatial structure modeling, as well as a phylogenomic approach. Initially, in order to elucidate the alginolytic potential of *Zobellia*, we calculated the content of polysaccharide lyase (PL) genes in each genome. The genus-specific PLs were PL1, PL6, PL7 (the most abundant), PL14, PL17, and PL40. We revealed that PL7 belongs to subfamilies 3, 5, and 6. They may be involved in local and horizontal gene transfer and gene duplication processes. Most likely, an individual evolution of PL7 genes promotes the genetic variability of the Alginate Utilization System across *Zobellia*. Apparently, the PL7 alginate lyases may acquire a sub-functionalization due to diversification between in-paralogs.

## 1. Introduction

Marine algal polysaccharides are an important nutrient source for marine bacteria. To utilize these polysaccharides, which significantly differ from terrestrial plant ones, marine bacteria have developed unusual degradation mechanisms. Key players in the degradation of complex polysaccharides are the marine *Flavobacteriia* of the phylum *Bac-teroidetes* [1,2]. The microorganisms feature different polysaccharide utilization loci (PULs), which encode a set of enzymes and other proteins involved in the breakdown of specific polysaccharides. The first recognized PULs were alginolytic operons associated with alginate utilization in marine *Bacteroidetes* [3]. The current studies of PULs are based on the sequencing of the genomes of cultured bacteria and metagenomes as a valuable resource of CAZymes and CAZyme machineries [4,5,6,7,8,9,10], allowing us to expand our knowledge of the bacterial degradation of algal polysaccharides.

The major bacterial players in marine polysaccharide degradation have become the subject of extensive research studies using genomic and transcriptomic approaches. Representatives of the genus *Zobellia* are marine *Flavobacteriia* and they specialize in algal polysaccharide degradation. The genus *Zobellia* was proposed by Barbeyron et al. (2001) [11]. First, the genus included two species: *Zobellia galactanivorans* and *Zobellia uliginosa*. Later, three new species were added [12]: *Zobellia russellii*, *Zobellia laminariae*, and *Zobellia amurskyensis*. To date, the five species of the genus *Zobellia* have validly published names (as listed at https://lpsn.dsmz.de/genus/zobellia, February 2021), and all originate from marine environments.

Members of the *Zobellia* genus are frequently found associated with red and brown algae [13]. *Z. galactanivorans* Dsij^T^, a marine species isolated from a red alga, was chosen as the type species [11]. Its genome exhibits a number of adaptive features such as consumption of algal polysaccharides, resistance to algal defense, and large amounts of CAZymes and sulfatases [14]. Numerous biochemical and structural studies have begun to unveil the complex enzyme systems for the degradation of various polysaccharides, such as agars, carrageenans, and laminarin [3,15,16,17,18,19,20,21].

In this work, we have applied genome-based approaches to investigate PL7 alginate lyases across the *Zobellia* genus.

## 2. Results and Discussion

### 2.1. Genomic Comparison of Zobellia Species

**Figure 1:**
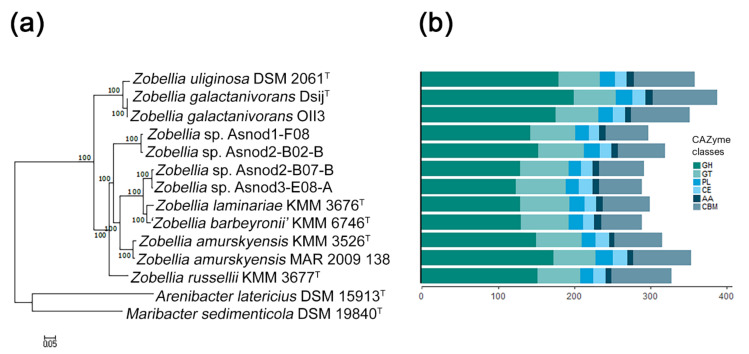
The phylogeny and CAZome of the genus *Zobellia*: (**a**) maximum-likelihood phylogeny based on 400 universal markers selected by PhyloPhlAn3.0 and reconstructed by RAxML with non-parametric bootstrapping using 100 replicates; (**b**) the bar plot showing the number of carbohydrate-active enzyme genes according to the CAZy classification for each strain. GH, glycoside hydrolase; GT, glycosyltransferase; PL, polysaccharide lyase; CE, carbohydrate esterase; AA, auxiliary activity; CBM, carbohydrate-binding module.

The characteristics of the ten publicly available *Zobellia* genomes and two unpublished ones (*Z. russellii,* KMM 3677^T^, and *Z. barbeyronii*, KMM 6746^T^, our data) were compared. Their genome sizes and GC content ranged from 4.92 to 5.52 Mb and from 36.7% to 42.8%, respectively. To clarify, the phylogenomic relationships of the *Zobellia* species, a phylogenetic tree of these genomes together with two other genomes of type strains from related genera, were built using PhyloPhlAn [22] based on 400 concatenated proteins. According to the genome tree (Figure 1a), all of the *Zobellia* strains clustered together, and the five subclades could be distinguished. The first subclade included a type strain of *Z. uliginosa* together with *Z. galactanivorans* strains, while other subclades consisted of type strains of *Z. laminariae* and ‘*Z. barbeyronii’*, as well as *Zobellia* sp. strains (Asnod2-F07-B and Asnod3-E08-A). The separated subclades were formed by strains of *Z. russellii*, *Z. amurskyensis* strains, and *Zobellia* sp. (Asnod1-F08 and Asnod2-F02-B). This clustering indicates a closer genome sequence similarity of the strains within subclades. Interestingly, the subclade *Z. uliginosa*/*Z. galactanivorans* was at the base of the *Zobellia* clade, implying an evolutionary divergence from other species of the genus.

Previous studies have revealed that the *Zobellia* genomes are abundant with CAZymes genes, which encode the ability to efficiently degrade complex polysaccharide substrates [14,23]. It has been known that carbohydrate degraders are characterized by a high proportion of CAZymes found in their genomes (more than 5% of the predicted protein-coding genes). To identify CAZymes in the *Zobellia* genomes, we used the dbCAN2 meta server (http://cys.bios.niu.edu/dbCAN2, December 2020) [24].

We found that in all *Zobellia* genomes CAZymes account for more than 6% (from 6.4% in *Zobellia* sp. Asnod3-E08-A to 7.6% in *Z. galactanivorans* Dsij^T^), which reflects the outstanding specialization of representatives of this genus in the degradation of polysaccharides. The total statistics of CAZyme classes predicted across the genomes are shown in Figure 1b. We found that *Z. galactanivorans* Dsij^T^ possesses the highest number of CAZymes (336), followed by *Z. amurskyensis* MAR 2009 138 (320), *Z. uliginosa* DSM 2061^T^ (315), and *Z. galactanivorans* OII3 (311). The smallest numbers of CAZymes were in *Zobellia* sp. Asnod3-E08-A (263) and Asnod2 B07BT (266). Notably, the genome of Z. *galactanivorans* Dsij^T^ encodes the largest and most diverse CAZYme repertoire, with approximately 60.7 CAZyme genes per Mb, in contrast to *Zobellia* sp. Asnod3-E08-A (52.5 CAZyme genes per Mb). However, these values indicate again a broad degradation potential conserved at the genus level [23].

**Figure 2:**
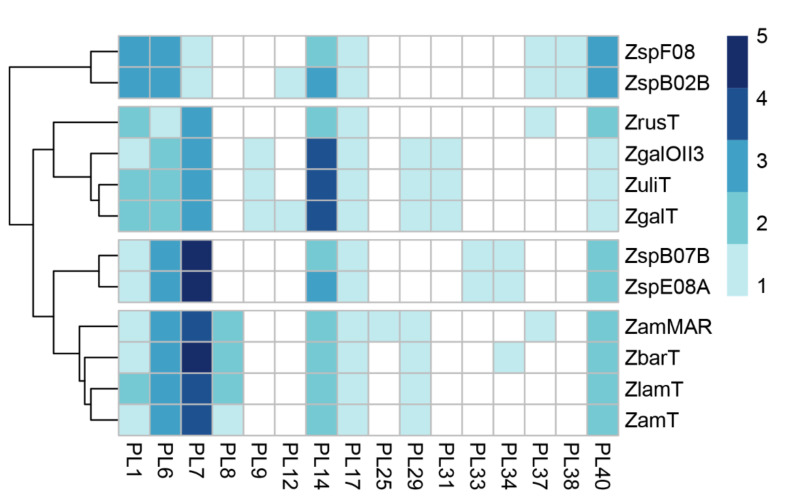
The distribution of polysaccharide lyase families across *Zobellia* genomes. The heat map shows the number of genes assigned to individual PL families. Rows are clustered using Euclidean distances. ZspF08, *Zobellia* sp. Asnod1-F08; ZspB02B, *Zobellia* sp. Asnod2-B02-B; ZrusT, *Z. russellii* KMM 3677^T^; ZgOII3, *Z. galactanivorans* OII3; ZulT, *Z. uliginosa* DSM 2061^T^; ZgaT, *Z. galactanivorans* Dsij^T^; ZspB07B, *Zobellia* sp. Asnod2-B07-B; ZspE08A, *Zobellia* sp. Asnod3-E08-A; Zammar, *Z. amurskyensis* MAR 2009 138; ZbarT, *Z. barbeyroni’* KMM 6746^T^; ZlamT, *Z. laminariae* KMM 3676^T^; ZamT, *Z. amurskyensis* KMM 3526^T^.

*Z. galactanivorans* is well known to degrade alginates of brown algae [25] due to its alginolytic system including alginate lyases of distinct polysaccharide lyase families. PLs are a group of enzymes that cleave uronic acid-containing polysaccharides via a β-elimination mechanism [26]. In order to elucidate the alginolytic potential of *Zobellia*, we calculated the content of the PL genes in these genomes (Figure 2). The heat map shows the frequency of the PL genes dedicated to PL families. Notably, the genomes of *Z. amurskyensis* MAR 2009 138 and Z. *galactanivorans* Dsij^T^ encode 25 and 24 PLs, while the genomes *Zobellia* sp. Asnod3-E08-A and Asnod2 B07BT encode only 17 and 16 PLs, respectively. Among the identified PLs, the genus-specific ones were PL1, PL6, PL7, PL14, PL17, and PL40. Since all *Zobellia* genomes contain PL genes of families PL6, PL7, PL14, and PL17, this indicates that they all have a functional alginate utilization pathway. PL7 is an important enzyme in the utilization of alginate. Surprisingly, PL7 genes are the most abundant PLs in *Zobellia* genomes, accounting for one to five copies (Figure 2).

### 2.2. PL7 Phylogenic and Structural Analyses

Alginate lyases from the PL7 family are widely distributed in bacteria and have a typical β-jelly roll fold, which can possess both endolytic and exolytic activities. To date, crystal structures of eleven PL7 algninate lyases have been elucidated, and at least 40 representatives were characterized from the PL7 family (CAZy database, February 2021). Based on the sequence similarity of catalytic domains, the family of PL7 has been subdivided into five subfamilies (SF1-SF5) [26]. Subfamily 6 (SF6) was proposed by Thomas et al. in the extensive study of the PL7 alginate lyases from *Z. galactanivorans* Dsij^T^ [21]. It has been suggested that PL7 enzymes from SF6 appear to be conserved only in marine representatives of the *Flavobacteriaceae*. Later, alginate lyase Aly7B_Wf from *Wenyingzhuangia funcanilytica* CZ1127^T^ was characterized and classified as belonging to subfamily SF6 of the PL7 family [27]. Recently, the crystal structure of a novel PL7 alginate lyase AlyC3 from *Psychromonas* sp. C-3 was reported [28]. The AlyC3, along with several other unclassified PL7 alginate lyases, was attributed to subfamily SF6, which implies belonging to the novel subfamily SF7. Thus, despite some confusion in the literature, subfamilies SF6 and SF7 could be distinguished in addition to the well-known subfamilies SF1-SF5.

**Figure 3:**
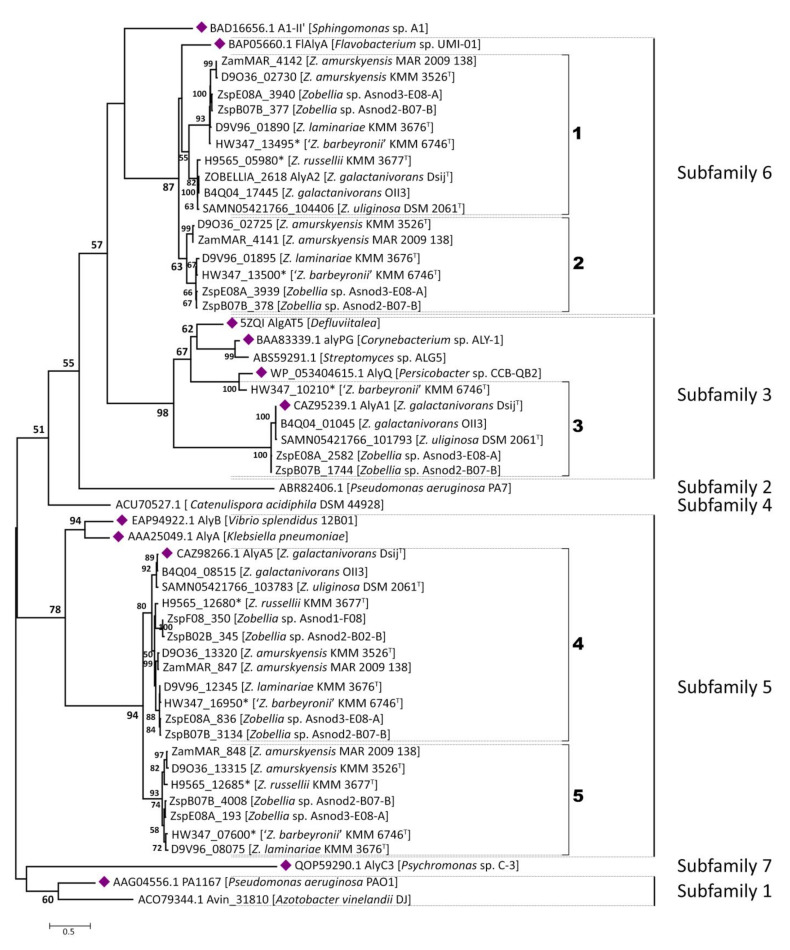
The phylogenetic tree of PL7 family alginate lyases from *Zobellia* and selected characterized representatives of PL7 family. For characterized PL7 family members, the corresponding GenBank accession numbers are given; for PL7 proteins of *Zobellia,* locus tags or RAST ORFs identifiers are listed. The organism names are listed in brackets. Proteins with a clarified crystal structure are marked as violet diamonds, and identifiers from unpublished genomes are marked with asterisks. Bootstrap values lower than 50 are not indicated.

Initially, to clarify the classification of predicted alginate lyases from *Zobellia* within the PL7 family, a phylogenetic tree was constructed with all the available characterized PL7 alginate lyases derived from CAZy database (data not provided). To avoid redundancy one more phylogenetic tree was obtained, which included only the most representative alginate lyases for each subfamily along with their target sequences (Figure 3).

In accordance with the ML tree, 41 PL7 alginate lyases from *Zobellia* fall strictly into SF3, SF5, and SF6. Although AlyA1 and AlyA5 from *Z. galactanivorans* Dsij^T^ have been biochemical and structural characterized in detail [21], it is worth noting that close inspections of orthologous and paralogous genes are of great value for investigation of the PL7 enzymes evolution at the genus level. For convenience, paralogs and orthologs in subclades were numbered from one to six on the phylogenetic tree.

It was revealed that only 6 of the 12 *Zobellia* representatives encode PL7 lyases belong to subfamily SF3—namely, *Z. galactanivorans* Dsij^T^, *Z. galactanivorans* OII3, *Z. uliginosa*, *Zobellia* sp. Asnod2-B07-B, *Zobellia* sp. Asnod3-E08-A, and ‘*Z. barbeyronii*’. Five of these, including AlyA1, were clustered together as presumptive orthologs, while ZbarT_PL7sf3_3 was reliably clustered with AlyQ from *Persicobacter* sp. CCB-QB2. We identified that the studied PL7-SF3 lyases had a modular organization and that all of them contain cleaved lipoprotein signals and CBM32 in addition to the catalytic domain. The same was determined earlier for AlyA1 [3]. Furthermore, domains moderately resembling CBM16 and CBM6 were also found in the architectures of AlyQ and ZbarT_PL7sf3_3, respectively.

The AlyA1 is an endolytic guluronate lyase [21], and AlyQ is most active on alginate, although it can also act on polyguluronate (poly-G) and polymannuronate (poly-M) [29]. For putative PL7-SF3 lyases from *Zobellia*, homology modeling based on the AlyA1 and AlyQ crystal structures was carried out (data not provided). The congruence of the phylogeny and structural similarities between these so-called orthologs indicate that they may possess similar activities. Considering that AlyA1 appears to have been acquired via horizontal gene transfer (HGT) from marine *Actinobacteria* [19], it becomes obvious that ZbarT_PL7sf3_3 was laterally acquired from other taxa. It is possible that CBM modules were fused with catalytic domains in ancestral genes before their transfer.

We consider that the PL7 alginate lyases belonging to subfamily SF5 are conserved within the *Zobellia* genus because at least one homolog was found in each genome. According to the phylogenetic tree, a well-supported (bpp = 94) orthologous group (OG) composed of 12 sequences is clearly distinguished and designated as 4 in Figure 3. Genes encoding putative PL7-SF5 lyases were duplicated and presented by paralogous pairs (marked as 5 on Figure 3) in seven *Zobellia* representatives—namely, *Z. russellii*, *Z. laminariae*, ‘*Z. barbeyronii*’, *Zobellia* sp. Asnod2-B07-B, *Zobellia* sp. Asnod3-E08-A, *Z. amurskyensis* KMM 3526^T^, and *Z. amurskyensis* MAR 2009 138. It should be noted that *Zobellia* sp. Asnod1-F08 and Asnod2-B02-B encode only one copy of the PL7 alginate lyases attributed to OG of the subfamily SF5. All the predicted PL7-SF5 lyases possess cleaved lipoprotein signals and PL7 catalytic domains. The amino acid sequence identities of PL7 catalytic domains between in-paralogous SF5_4 and SF5_5 were in the range of 65.0% to 67.51%. The identities between AlyA5 from *Z. galactanivorans* Dsij^T^ and orthologs from OG SF5_4 varied within the range of 83.16% to 98.97%, while they were in the range of 64.98% to 66.79% across the AlyA5 and out-paralogs from SF5_5 (Table S2).

**Figure 4:**
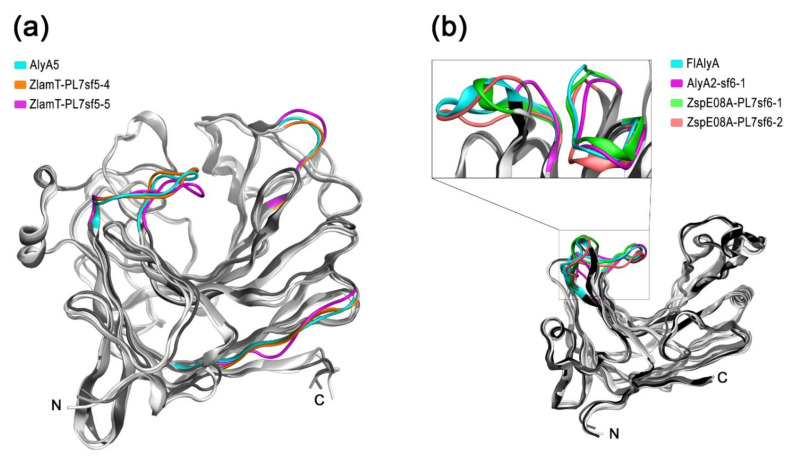
The superimposition of paralogous alginate lyases PL7 from SF5 and SF6 to known structures: (**a**) ribbon representation of the superimposition of the predicted 3D models for paralogous PL7-SF5_4 and SF5_5 from *Z. laminariae* KMM 3676^T^ onto prototype AlyA5 from *Z. galactanivorans* Dsij^T^ (PDB code 4BE3); (**b**) ribbon representation of the superimposition of predicted 3D models for paralogous PL7-SF6_1 and SF6_2 from *Zobellia* sp. Asnod3-E08-A and AlyA2 SF6_1 from *Z. galactanivorans* Dsij^T^ onto prototype FlAlyA from *Flavobacterium* sp. UMI-01 (PDB code 5Y33). Ribbon representations of superimpositions are presented in shades of grey. Differences between spatial structures of the paralogous alginate lyases in types of external loops are shown in color. The color corresponding to each structure is indicated.

In comprehensive study, it was determined that AlyA5 cleaves monomers from the non-reducing end of oligoalginates in an exolytic fashion [21]. The three-dimensional structures of in-paralogous ZlamT_PL7sf5_4 and ZlamT_ PL7sf5_5 were modelled using AlyA5 (PDB ID: 4BE3) as the template and superimposed. Based on structural alignment (Figure S1), the main divergences between the catalytic domains of the ortholog and paralogs were colored in ribbon representation (Figure 4a). It was revealed that the orthologs from OG SF5_4 shared a closer similarity in 3D structures to each other in contrast to in-paralogs, which confirms the conclusions following from the pairwise sequence identity analysis. Although the overall fold of *Zobellia* representatives PL7-SF5 is mostly similar, particularly in terms of the catalytic groove, there are slight differences in the external loop configurations.

The most curious and puzzling insights were obtained regarding the PL7 enzymes belonging to the subfamily SF6. It was revealed that 10 of 12 *Zobellia* representatives encode PL7-SF6 lyases, except *Zobellia* sp. Asnod1-F08 and Asnod2-B02-B. A suppositive OG for subfamily SF6 was distinguished (bpp = 55) and designated as one at the phylogenetic tree (Figure 3). In the same representatives, except *Z. russellii*, these lyases were presented by in-paralogs, as was revealed for subfamily SF5. All putative PL7-SF6 lyases contain only cleaved lipoprotein signals along with PL7 catalytic domains. Pairwise identities, calculated for as in-paralogs (59.92–61.54%), AlyA2 versus alleged orthologs (66.80–100%), and out-paralogs (64.78–68.83%, Table S2), were insufficient for reliable delineation of orthologous group in SF6 because obtained values did not agreed with generally accepted criteria.

To date, among the PL7 alginate lyases from subfamily SF6, only two enzymes have been studied. The Aly7B_Wf from *W. funcanilytica* CZ1127^T^ was characterized as endo-acting bifunctional alginate lyase and preferably cleaved polyM [27]. The FlAlyA from *Flavobacterium* sp. UMI-01 was first characterized as an endolytic enzyme with a preference for polymannuronate [30], and later, its crystal structure was clarified [31]. The three-dimensional structures of the orthologous AlyA2, and in-paralogous PL7-SF6 from *Zobellia* sp. Asnod3-E08-A, were modeled using FlAlyA (PDB ID: 5Y33) as the template and superimposed. The most significant divergences between 3D structures were colored in ribbon representation (Figure 4b). The overall fold of *Zobellia* PL7-SF6 lyases was mostly matched to prototype structure, but there were moderate differences in external loop configurations, which may imply the sub-functionalization of PL7-SF6. Considering the observed peculiarities of 3D structures, which are reflected in structural alignment (Figure S2), it has become clear that the PL7-SF6 are characterized by high diversification within the *Zobellia* genus.

A detailed exploration of genetic loci containing genes for PL7 lyases may shed light on the debatable issue regarding both the OGs delineation and the role of gene duplication.

### 2.3. Comparative Analysis of PL7-Containing Loci between Zobellia Genomes

The marine flavobacterium *Z. galactanivorans* Dsij^T^ constitutes a model organism for studying algal polysaccharide bioconversions, including alginates [14]. For the first time, the Alginate Utilization System (AUS) for marine bacterium has been identified in *Z. galactanivorans* Dsij^T^ and studied in detail [3]. One more comprehensive investigation regarding alginate utilization loci was carried out for the marine ‘*Gramella forsetii*’ KT0803 [6]. Recently, it was reported that key enzymes for alginate utilization are widespread across 60 strains, which were isolated from marine environments and belong to the phyla *Proteobacteria* and *Bacteroidetes* [7].

According to the literature, in *Z. galactanivorans* Dsij^T^ the AUS is encoded by two operons and two genes isolated in the genome [14]. The activity of the system is tightly controlled by the presence of alginate in the medium [32] and AusR, a GntR family repressor [33]. As described in [33], the current model of alginate degradation by *Z. galactanivorans* Dsij^T^ implies stepwise depolymerization of alginate by coherent action of extracellular lyases AlyA1 (PL7) and AlyA7 (PL14) then oligosaccharides, recruited by surface-exposed PKD-containing and SusD-like proteins, are imported to the periplasm via TBDT where they are subjected to further degradation into unsaturated mono-uronic acid by the alginate lyases AlyA2 (PL7), AlyA3 (PL17), AlyA4 (PL6), AlyA5 (PL7) and AlyA6 (PL6). Further conversions in the cytoplasm occur through the consecutive action of KdgF, SDR, and KdgK1. Finally, KDPG (2-keto-3-deoxy-6-phosphogluconate) is eventually assimilated into the central metabolism through the Entner-Doudoroff pathway.

**Figure 5:**
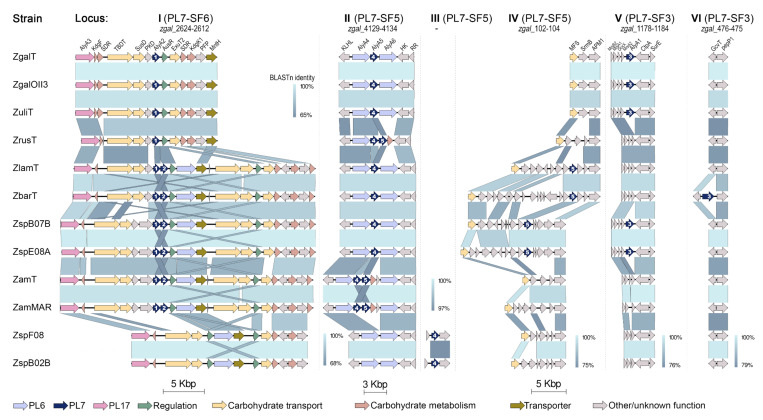
The comparisons of six loci (I–VI) containing PL7 genes among the *Zobellia* representatives. Genes were colored based on their annotation as indicated at the bottom of the figure; ZgaT, *Z. galactanivorans* Dsij^T^; ZgOII3, *Z. galactanivorans* OII3; ZulT, *Z. uliginosa* DSM 2061^T^; ZrusT, *Z. russellii* KMM 3677^T^; ZlamT, *Z. laminariae* KMM 3676^T^; ZbarT, ‘*Z. barbeyronii’* KMM 6746^T^; ZspB07B, *Zobellia* sp. Asnod2-B07-B; ZspE08A, *Zobellia* sp. Asnod3-E08-A; ZamT, *Z. amurskyensis* KMM 3526^T^; Zammar, *Z. amurskyensis* MAR 2009 138; ZspF08, *Zobellia* sp. Asnod1-F08; ZspB02B, *Zobellia* sp. Asnod2-B02-B.

In order to clarify the issues mentioned above, we performed a comparative analysis of loci containing PL7 genes across the *Zobellia* genomes (Figure 5).

It was revealed that PL7 genes are localized in six genetic loci (I–VI), which are part of a complex and evolved AUS, in agreement with previously those defined for the AlyA1, AlyA2, and AlyA5 from *Z. galactanivorans* Dsij^T^ [14]. Thus, the trend toward the distribution of PL7 alginate lyases genes over separate loci is conserved within the *Zobellia* genus. It is noteworthy that PL7 of subfamilies SF3 and SF6 are encoded strictly in separate loci V or VI, and I, respectively. Whereas, in-paralogous genes from PL7-SF5 might be located adjacently in locus II and separately in loci II, III, and IV.

As presented on the synteny plot, the orthologs PL7-SF3 from *Z. galactanivorans* Dsij^T^, *Z. galactanivorans* OII3, *Z. uliginosa*, *Zobellia* sp. Asnod2-B07-B and Asnod3-E08-A were encoded in locus V in the same surroundings, whereas orthologs from ‘*Z. barbeyronii*’ was collocated within another environment in locus VI. This confirms that all of them were laterally acquired, in agreement with phylogenetic analysis points. It can be assumed that such dispersed genes are plastic in genomes and more frequently participate in HGTs processes.

Ten PL7 genes from OG SF5_4 are collocated along with PL6 lyases genes in locus II, which was previously described in *Z. galactanivorans* Dsij^T^ genome as small operon *zgal*_4130-4132 [3]. Seven PL7-SF5 genes are presented by in-paralogs; among them, three SF5_5 paralogs are collocated with SF5_4 in locus II (in *Z. russellii*, *Z. amurskyensis* KMM 3526^T^, *Z. amurskyensis* MAR 2009 138), whereas four other SF5_5 paralogs are in different locus IV (in *Z. laminariae*, ‘*Z. barbeyronii*’, *Zobellia* sp. Asnod2-B07-B and Asnod3-E08-A). Interestingly, SF5_4 genes of *Zobellia* sp. Asnod1-F08 and Asnod2-B02-B are placed in locus III, which is not represented in *Z. galactanivorans* Dsij^T^ genome. We suppose that the processes of gene duplication and local gene transfer represent the evolution of PL7-SF5 genes at the *Zobellia* genus level. Summarizing insights from phylogenetic and synteny analyses, in the case of SF5 in-paralogs colocation, the SF5_4 from the orthologous group is located upstream. Surprisingly, the pairwise identities of PL7 catalytic domains between SF5_4 and SF5_5 remained about 66% on average, regardless of whether they collocated or not.

Altogether, except genomes of *Zobellia* sp. Asnod1-F08 and Asnod2-B02-B, all the genomes contain the PL7-SF6 orthologs, localized in a large operon (locus I) along with other carbohydrate-related genes. The operon *zgal*_2624-2612 containing AlyA2 from *Z. galactanivorans* Dsij^T^ was described in detail [3]. Six PL7-SF6 genes are presented by in-paralogs, for which upstream localization is a reliable criterion for belonging to OG PL7-SF6, as was identified for PL7-SF5 and discussed above. A hypothesized sub-functionalization of in-paralogs SF6_1 and SF6_2 is supported by the presence of additional non-paralogous transporters and PKD-containing protein genes, as well as by the duplication of the repressor *AusR*, at the locus I. Our highlight of the variations in PL lyases content is another interesting observation based on synteny analysis of locus I. The first type of locus I contains genes for PL17 and PL7-SF6_1 (strains of *Z. galactanivorans*, *Z. uliginosa*, and *Z. russellii*); the second one comprises PL17, PL7-SF6 in-paralogs, and PL6 (*Z. laminariae*, ‘*Z. barbeyronii*’, *Zobellia* sp. Asnod2-B07-B and Asnod3-E08-A, strains of *Z. amurskyensis*); the third type contains PL17 and PL6 (*Zobellia* sp. Asnod1-F08 and Asnod2-B02-B).

## 3. Materials and Methods

### 3.1. Phylogenomic Analysis

Representative genomes for *Zobellia* and related genera were retrieved from GenBank, NCBI. Accession numbers for the genomes used in this study are provided in Table S1. Single-copy marker genes (*n* = 400) were selected from protein sequences, concatenated, and aligned using the PhyloPhlAn (version 3.0) pipeline [22]. A maximum-likelihood tree was reconstructed by RAxML (version 8.2.12) under the LG+Γ model with non-parametric bootstrapping using 100 replicates [34].

### 3.2. Annotation of Carbohydrate-Active Enzymes

The carbohydrate-active enzymes and carbohydrate-binding modules were annotated using the dbCAN2 Meta server (version 9.0) with default settings [24]. Predictions by at least one of the three algorithms integrated within the server (DIAMOND, HMMER, and Hotpep) were considered sufficient for CAZy family assignments. The relative abundances of CAZymes and PLs were visualized by stacked bar plots and heat maps using the ggplot2 (version 3.3.3) and pheatmap (version 1.0.12) packages in RStudio (version 1.3.1093) with R (version 4.0.3, R Foundation for Statistical Computing) [35,36].

### 3.3. Sequence Analyses and Homology Modelling

The PL7 lyases were inspected for the presence of additional functional domains via the Interproscan server (version 83.0) [37]. For phylogenetic analysis protein sequences of 11 alginate lyases with solved structures belonging to the PL7 family were selected in the CAZy database [38]. Multiple sequence alignment of PL7 catalytic domains was performed using ClustalX implemented in MEGA-X (version 10.1.8) and manually corrected [39]. A maximum-likelihood phylogeny with 100 non-parametric bootstrap replicates was calculated using the IQ-TREE web server (version 1.6.12) and the WAG+I+G4 substitution model determined using ModelFinder [40,41].

The pairwise sequence identities were calculated by Clustal (version 2.1) using EMBL-EBI Services [42]. The 3D-structures of paralogous PL7 alginate-lyases from SF3, SF5, and SF6 were generated using homology modeling by the Molecular Operating Environment (MOE, version 2020.09) [43]. The crystal structures of the AlyA1 and AlyA5 from *Z. galactanivorans* Dsij^T^, AlyQ from *Persicobacter* sp. CCB-QB2, and FlAlyA from *Flavobacterium* sp. UMI-01 with PDB codes 3ZPY, 4BE3, 5XNR, and 5Y33, respectively, were used as the initial templates. Structure-based sequence alignments were visualized with ESPript 3 [44].

### 3.4. Comparative Analysis of PL7-Containing Loci

The genomic regions containing PL7 genes were extracted from the GBK files of the *Zobellia* genomes using Geneious Pro software (version 4.8) [45]. Due to the lack of genome annotations for *Zobellia* spp. and *Z. amurskyensis* MAR 2009 138, their genomes were initially annotated by the RAST server [46]. Identifiers for PL7 and adjacent genes included in selected loci are listed in Table S3. Generated GBK files were modified by adding custom color feature qualifiers. Pairwise comparisons of each locus between twelve genomes were carried out using BLASTn (BLAST version 2.11.0+) run in EasyFig (version 2.2.5) [47]. Synteny plots were visualized by Easyfig with the minimum BLAST hit of 680 bp.

## 4. Conclusions

We revealed that PL7 family alginate lyases are the most abundant among the polysaccharide lyases identified in *Zobellia* genomes. Based on phylogenomic, structural, and comparative analyses, the PL7 lyases belong to SF3, SF5, and SF6 and are involved in local and horizontal gene transfer, as well as gene duplications processes. It is most likely that an individual evolution of PL7 genes may promote Alginate Utilization System variability across the *Zobellia* genus. The PL7 alginate lyases may acquire a sub-functionalization due to diversification between in-paralogs.

## Data Availability

Unpublished genomes are available from the authors.

## Supplementary Materials

Figure S1: Structure-based sequence alignment of PL7 alginate lyases from subfamily SF5; Figure S2: Structure-based sequence alignment of PL7 alginate lyases from subfamily SF6; Table S1: List of accession numbers for genomes used in the phylogenomic analysis; Table S2: Identity matrix of catalytic domains from PL7 alginate lyases identified in *Zobellia* genomes; Table S3: Identifiers of PL7 and adjacent genes included in loci I–VI.
