# Supplementary material for: Genome-Wide Analysis of PL7 Alginate Lyases in the Genus Zobellia
Source: Molecules. 2021 Apr 20;26(8):2387. doi: 10.3390/molecules26082387 (PMC8073546; doi:10.3390/molecules26082387)
Supplement: Supplementary file 1 [file molecules-26-02387-s001.zip › Supplementary_file_v2.pdf]

Supplementary materials

“Genome wide analysis of PL7 family alginate lyases in the genus *Zobellia*”

Nadezhda Chernysheva, Evgeniya Bystritskaya, Galina Likhatskaya, Olga Nedashkovskaya, and Marina Isaeva *

G.B. Elyakov Pacific Institute of Bioorganic Chemistry, Far Eastern Branch, Russian Academy of Sciences, 159, Pr. 100 let Vladivostoku, Vladivostok 690022, Russia; chernysheva.nadezhda@gmail.com (N.C.); belyjane@gmail.com (E.B.); galin56@mail.ru (G.N.); oned2004@mail.ru (O.N.)

* Correspondence: issaeva@gmail.com (M.I.); Tel.: ++7-423-231-1168

Submitted to Molecules.

Table S1. List of accession numbers for genomes used in phylogenomic analysis.

Strain	GenBank Accession No.
Zobellia uliginosa DSM 2061^{T}	FTOB00000000.1
Zobellia galactanivorans DsiJ^{T}	FP476056.1
Zobellia galactanivorans OII3	MWRZ00000000.1
Zobellia sp. Asnod1-F08	JADDXT000000000.1
Zobellia sp. Asnod2-B02-B	JADDXS000000000.1
Zobellia sp. Asnod2-B07-B	JADDXR000000000.1
Zobellia sp. Asnod3-E08-A	JADDXQ000000000.1
Zobellia laminariae KMM 3676^{T}	RCNS00000000.1
'*Zobellia barbeyronii*' KMM 6746^{T}	JACATN000000000.1*
Zobellia amurskyensis KMM 3526^{T}	RCNR00000000.1
Zobellia amurskyensis MAR 2009 138	JQMD00000000.1
Zobellia russellii KMM 3677^{T}	JACSOI000000000.1*
Arenibacter latericius DSM 15913^{T}	AUKX00000000.1
Maribacter sedimenticola DSM 19840^{T}	FZNV00000000.1

*Unpublished data

Table S2. Identity matrix of catalytic domains from PL7 alginate lyases identified in *Zobellia* genomes.
Table S3. Identifiers of PL7 and adjacent genes included in loci I–VI.

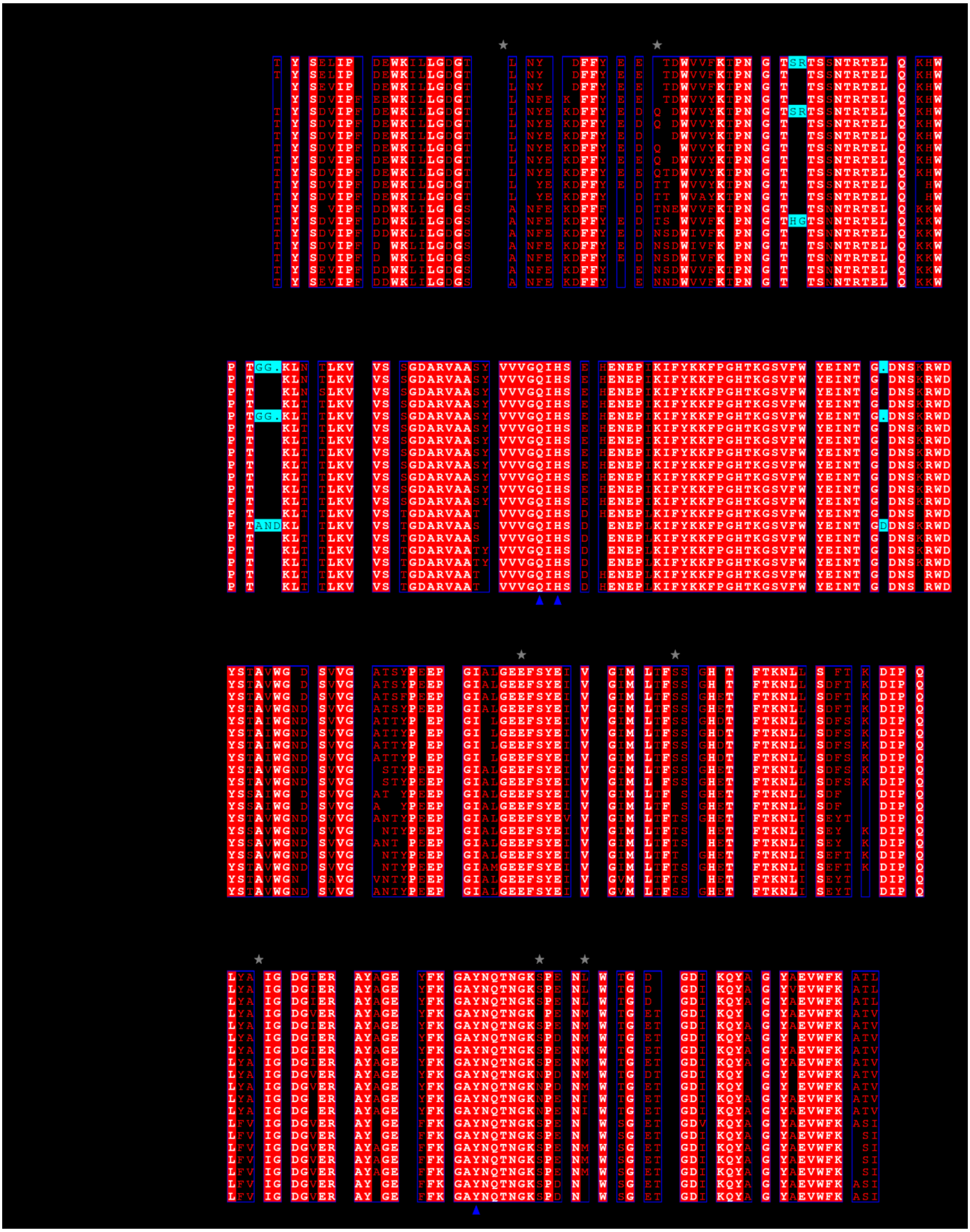

Figure S1. Structure-based sequence alignment of PL7 alginate lyases from subfamily SF5. The sequences are aligned using known structure, AlyA5 from *Z. galactanivorans* Dsij^{T} (PDB ID: 4BE3). The secondary structures are shown above the alignment. Conserved amino acids in white letters highlighted on the red background are identical and those in red letters are similar. α-Helices are represented as schematically, and β-turns are marked with *TT*. Blue triangles indicate the conserved residues involved in the catalytic machinery. The differences between in-paralogs, which were colored in ribbon representations of superimpositions are highlighted by cyan background.

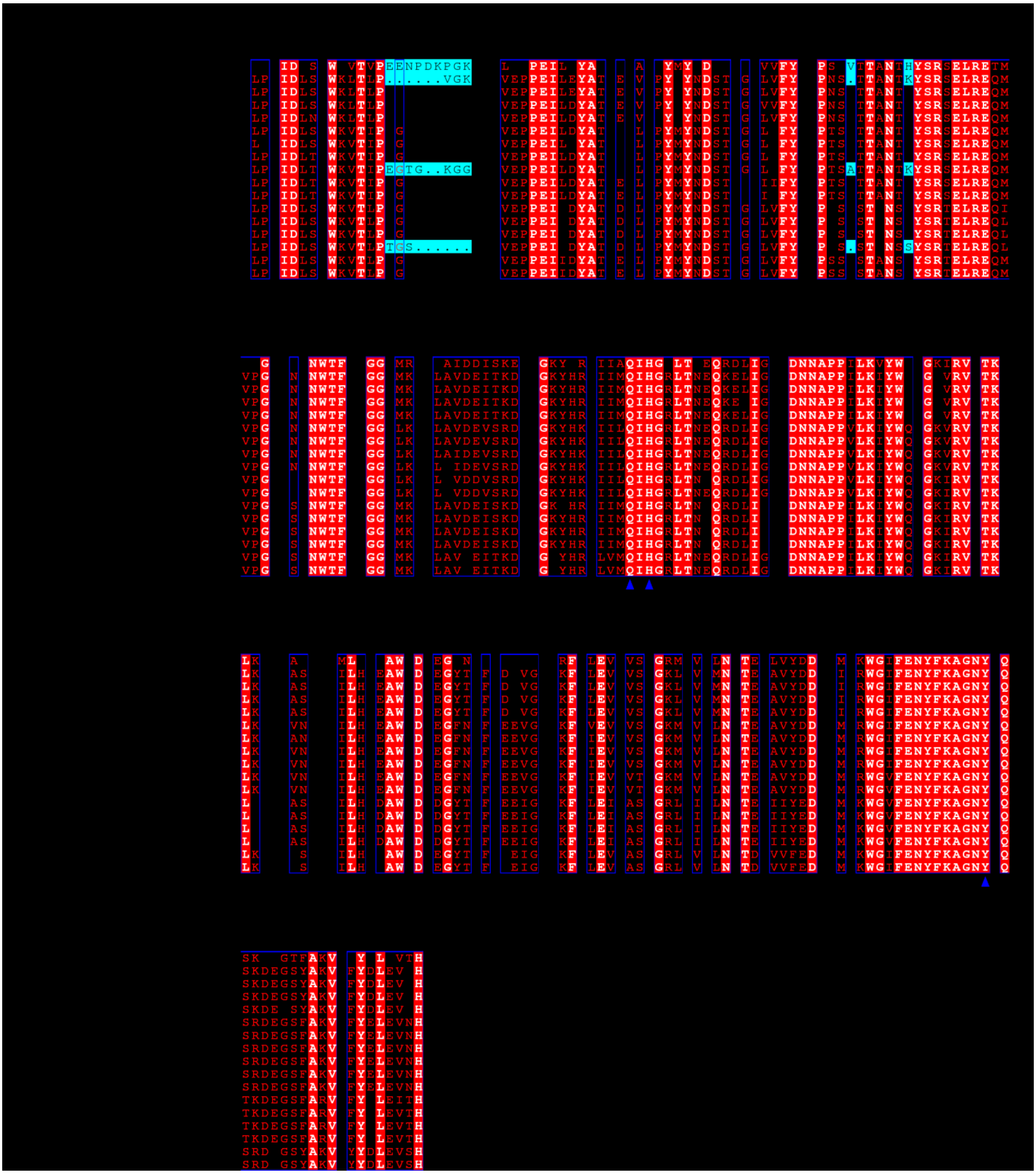

Figure S2. Structure-based sequence alignment of PL7 alginate lyases from subfamily SF6. The sequences are aligned using known structure, FlAlyA from *Flavobacterium* sp. UMI-01 (PDB ID: 5Y33). The secondary structures are shown above the alignment. Conserved amino acids in white letters highlighted on the red background are identical and those in red letters are similar. α-Helices are represented as schematically, and β-turns are marked with *TT*. Blue triangles indicate the conserved residues involved in the catalytic machinery. The differences between in-paralogs, which were colored in ribbon representations of superimpositions are highlighted by cyan background.
