# Supplementary figures and images for: Genome-Wide Analysis of PL7 Alginate Lyases in the Genus Zobellia
Source: Molecules. 2021 Apr 20;26(8):2387. doi: 10.3390/molecules26082387 (PMC8073546; doi:10.3390/molecules26082387)

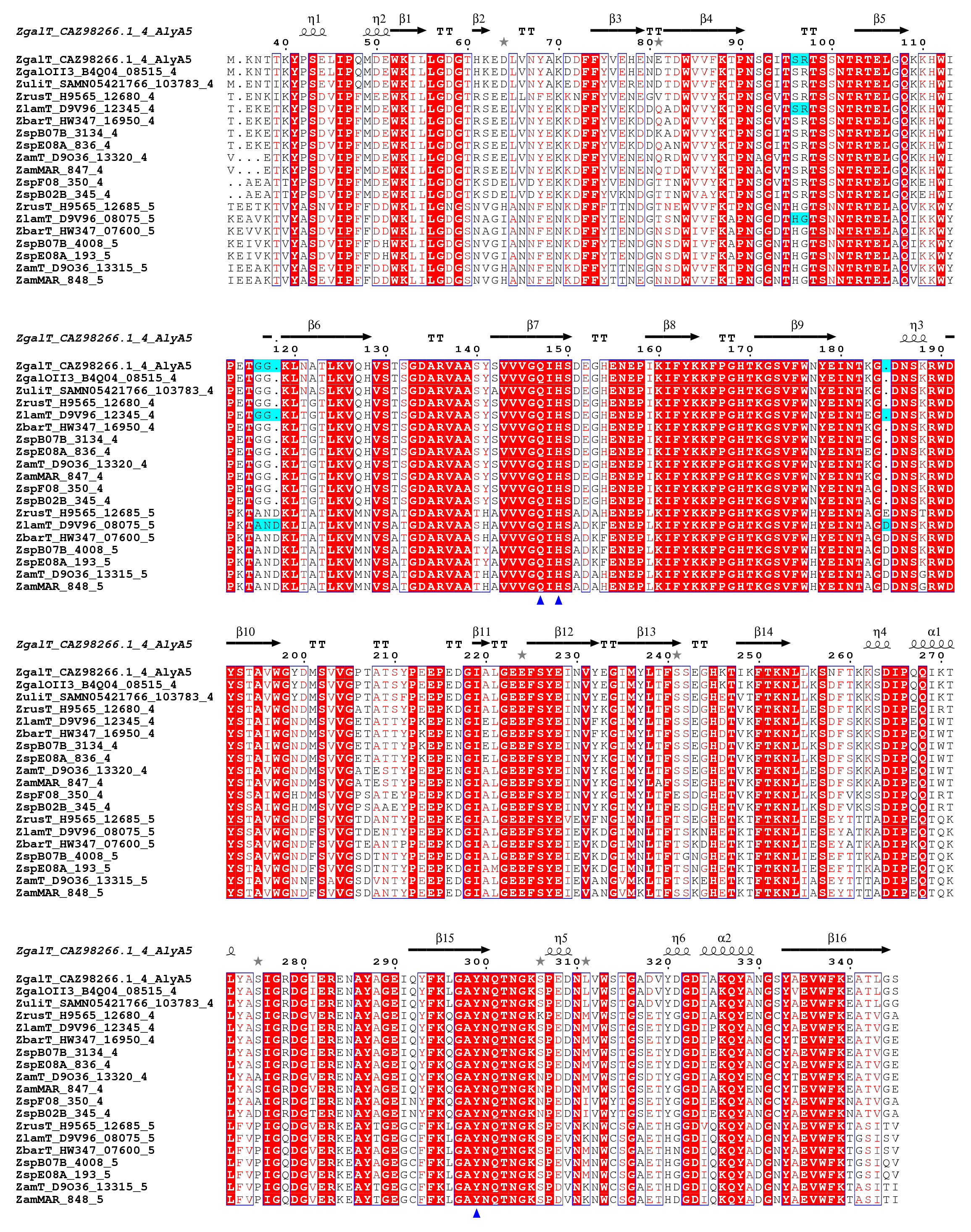

Supplement: Supplementary file 1 [file molecules-26-02387-s001.zip › FigS1_SF5_v2.tiff]

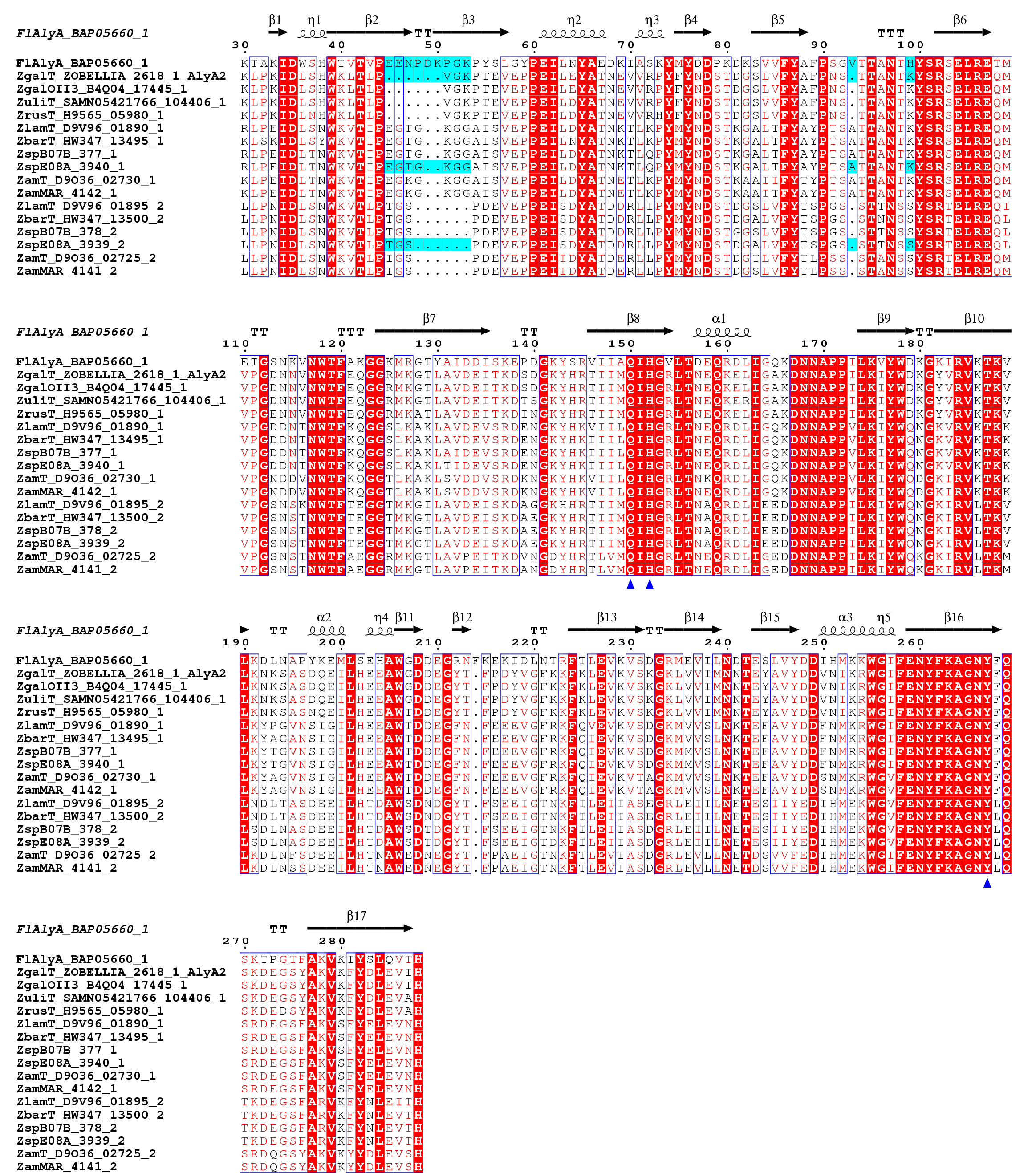

Supplement: Supplementary file 1 [file molecules-26-02387-s001.zip › FigS2_SF6_v2.tiff]
